# Transcriptomic Data Analyses Reveal a Reprogramed Lipid Metabolism in HCV-Derived Hepatocellular Cancer

**DOI:** 10.3389/fcell.2020.581863

**Published:** 2020-10-27

**Authors:** Guoqing Liu, Guojun Liu, Xiangjun Cui, Ying Xu

**Affiliations:** ^1^School of Life Sciences and Technology, Inner Mongolia University of Science and Technology, Baotou, China; ^2^Cancer System Biology Center, The China-Japan Union Hospital of Jilin University, Changchun, China; ^3^School of Natural Sciences and Mathematics, Ural Federal University, Yekaterinburg, Russia; ^4^Computational Systems Biology Laboratory, Department of Biochemistry and Molecular Biology, and Institute of Bioinformatics, University of Georgia, Athens, GA, United States

**Keywords:** hepatocellular cancer, lipid metabolism, *de novo* synthesis of fatty acid, gene set enrichment, gene co-expression

## Abstract

Reprograming lipid metabolism, one of the major metabolic alterations in cancer, is believed to play an essential role in cancer development, but the exact molecular mechanism remains elusive. Here, we present a computational study of transcriptomic data of HCC with HCV etiology to investigate how lipid metabolism alters during HCC progression. Our analyses reveal that: (1) cancer tissue cells tend to synthesize fatty acids *de novo* and its phospholipid derivatives; (2) lipid catabolism and fatty acid oxidation are remarkably down-regulated in HCC; (3) the lipid metabolism in HCC is largely independent of lipids in blood circulation; (4) stage-specific co-expression networks for lipid metabolic genes were identified during HCC progression; and (5) the expression levels of several lipid metabolic genes that are differentially expressed or co-expressed specifically at the HCC stage have a strong correlation with cancer survival. Overall, the results provide detailed information about the reprogramed lipid metabolism in HCV-derived HCC.

## Introduction

Liver cancer is the third leading cause of cancer-related death worldwide, and has had approximately 840,000 new cases per year in the recent history (Bray et al., 2018). Hepatocellular cancer (HCC) accounts for 90% of the primary liver cancer cases, and cholangiocarcinoma, derived from the epithelial lining of the bile duct, is a distant second and rare (Llovet et al., 2016). A few risk factors have been identified for HCC, such as viral infection, alcohol over dose, smoking, ingestion of aflatoxin B1, and non-alcoholic steatohepatitis. Infection with hepatitis B virus (HBV) and hepatitis C virus (HCV) is a major risk factor for HCC worldwide. For example, up to 80% of HCC is attributable to HBV or HCV (Perz et al., 2006).

Hepatocellular cancer development generally consists of a few stages, namely chronic inflammation, advanced hepatic fibrosis or cirrhosis, dysplastic nodules, and HCC (Llovet et al., 2016). Of all HCC cases, 80–90% have cirrhosis prior to cancer development (Caldwell and Park, 2009), largely due to chronic inflammation for an extended period of time. It was estimated that 20–30% of people with HBV and HCV infection will develop cirrhosis 20–30 years after the infection. Only a small portion of HCC with HBV or HCV etiology developed without cirrhosis.

The molecular mechanism of the formation and progression of HCC remains elusive, although great progress has been made. Considerable genomic alterations induced by hepatitis virus infection have been believed to contribute to the hepatocyte malignant transformation through various oncogenic signaling pathways, such as Wnt/β-catenin, PI3K/AKT/mTOR, TGF-β, insulin-like growth factor receptor (IGFR), and MAPK signaling pathways (Bruix et al., 2015; Li and Wang, 2016; Llovet et al., 2016). Driver mutations in HCC have also been shown to affect the inactivation of p53, chromatin remodeling, alternative splicing, and the induction of the oxidative stress response pathways, which may play a role in carcinogenesis and cancer progression (Ralph et al., 2010; Bruix et al., 2015; Pon and Marra, 2015; Climente-González et al., 2017).

Metabolic reprograming has a tight and complex relationship with genetic mutations in cancerous cells. Although multiple mutations along with the dysregulation of various signaling pathways have long been considered as a major molecular cause of tumorigenesis, metabolic alterations triggered by the oncogenic mutations and signaling, which are probably preferentially selected by the pre-cancerous or cancerous cells to survive in the stressful cellular microenvironment, are now shown to play much more critical roles than previously thought in cancer development (Cairns et al., 2011). It is even possible that some metabolic alterations occur before oncogenic signals and drive malignant transformation.

Metabolic reprograming in cancer includes alterations in the metabolisms of carbohydrates, lipids, and amino-acids (Zhang and Du, 2012; Yang et al., 2013; Hirschey et al., 2015). The dysregulation of lipid metabolism is one of the major reprogramed metabolisms in cancer. The most studied lipid metabolic reprograming in cancer is the elevated *de novo* synthesis of fatty acids and downstream lipogenesis (Zhang and Du, 2012). Like proliferative embryonic cells, cancer cells prefer to synthesize *de novo* fatty acids for use rather than importing them from circulation as normal somatic cells do. It has been reported that the increased level of fatty acids in cancer cells will be esterified to lipids (e.g., phospholipids), which can be used as building blocks in the cell membrane or for energy production via β-oxidation (Swinnen et al., 2006; Menendez and Lupu, 2007; Zhang and Du, 2012). In addition, such lipids can also serve as signaling molecules such as ceramide (a sphingolipid). It is noteworthy that cancer cells tend to simultaneously up-regulate their lipid synthesis and degradation processes (Nomura et al., 2010; Zhang and Du, 2012).

The altered lipid metabolism is now believed to contribute to cancer initiation and progression (Santos and Schulze, 2012; Baenke et al., 2013; Beloribi-Djefaflia et al., 2016; Corbet and Feron, 2017; Cheng et al., 2018), but the exact molecular mechanisms are not fully understood yet due to the high complexity and temporal spatial heterogeneity of tumors. In addition, the reprogramed lipid metabolism seems to not function alone, instead it is coupled with other reprogramed metabolisms. For example, increased lipid synthesis generally requires changes in glucose and/or amino acid metabolisms, which may serve as the substrates for fatty acid synthesis via citric acid, or increased utilization of β-oxidation may divert glucose and amino acids toward a purpose other than ATP production.

Here, we present a computational analysis of gene expression data of HCV-related HCC tissues to address the following questions: (1) How do the lipid metabolic genes (LMGs) change in their expressions during HCC formation and reprogram the lipid metabolism in cancer? (2) What signatures do the co-expression network for lipid metabolism-related genes have and how does the network evolve as the disease advances through different stages? (3) Are there any LMGs correlated with cancer survival?

## Materials and Methods

### Gene Expression Data

The microarray gene-expression data of 75 liver tissue samples derived from GEO (GSE6764) were analyzed in this study. This dataset consists of 10 normal tissue samples and 65 disease samples collected from patients with HCV infection: 13 cirrhotic tissue samples, 17 dysplastic nodules, and 35 HCC samples.

### Methods

#### Identification of Differentially Expressed Genes

The microarray raw data were normalized by using RMA (Irizarry et al., 2003). For the genes with multiple probes only those with the largest mean of expression across the total samples were retained. The final dataset consists of the expression of 22,880 genes that have official gene symbols. Differential gene expression between disease sample sets (cirrhosis, dysplasia, and HCC) and a normal sample set were determined using limma (Ritchie et al., 2015; Phipson et al., 2016), in which the Benjamini-Hochberg (BH) method was used for multiple testing. Differentially expressed genes (DEG) are defined as those meeting two criteria: adjusted *p*-value <0.01 and | log_2_FC| > 1, where FC denotes a fold change.

#### Gene Set Enrichment Analysis

Gene set/pathway enrichment analysis was done against GO terms (Ashburner et al., 2000; The Gene Ontology Consortium, 2017) using ClusterProfiler (Yu et al., 2012) with minor modifications: (1) the DEGs in each disease sample set were used as a query gene set, and *p*-values were computed separately for the up-regulated and down-regulated genes using the hypergeometric test; (2) if a GO term or KEGG pathway was enriched by both up-regulated and down-regulated genes (“BH” adjusted *p*-value <0.01), the term with the smaller *p*-value was used; (3) for an enriched pathway, if the fraction of the up-regulated genes (or down-regulated genes) in the DEGs mapped to the pathway was at least 60%, we considered the pathway “activated” (or repressed).

#### Lipid Metabolic Genes

LMGs refer to genes that are involved in the lipid metabolism, which were obtained from GO^1^. LMGs in the following GO terms were analyzed in this study (Supplementary Table S1): fatty acid biosynthesis (GO: 0006633), β oxidation (GO: 0006635), lipid biosynthetic process (GO: 0008610), lipid catabolic process (GO: 0016042), lipid metabolic process (GO: 0006629), cholesterol metabolic process (GO: 0008203), cholesterol transport (GO: 0030301), and lipid transport (GO: 0006869). The integrated LMG set had 1,619 unique genes.

#### Consistently Regulated LMGs and Stage-Specific LMGs

Consistently up-regulated (or down-regulated) LMGs were defined as those up-regulated (or down-regulated) in all stages vs. control samples. Stage-specific LMGs were those satisfying the following criteria: (1) the genes were up-regulated (or down-regulated) at a specific disease stage vs. control samples, but either down-regulated (or up-regulated) or not differentially expressed at all other stages; and (2) the genes were differentially expressed at a specific disease stage as compared with each of the other stages.

#### Inference of Gene Co-Expression Network

A pair of genes were considered as co-expressed if their expression levels across the specified samples had a strong correlation, namely Pearson’s correlation: | *R*| > 0.5 and “BH” adjusted *p*-value <0.05.

#### Stage-Specific Co-expression Networks

We considered a co-expression network as specific to a particular stage of liver disease, namely normal, cirrhosis, dysplastic or HCC, if each pair of co-expressed genes in the network (1) was specific to the disease stage; and (2) its correlation type (positive vs negative) was different in this stage from those in all the other stages. The co-expressed genes were visualized using Cytoscape (Shannon et al., 2003).

#### Survival Analysis

Survival analysis was performed by using GEPIA (Tang et al., 2017), which is based on TCGA expression data. The Liver Hepatocellular Carcinoma (LIHC) samples were divided into two groups based on the expression levels of a specific gene of interest (group cutoff: median), and a log-rank test was used to test the difference in the overall survival between the two groups.

## Results and Discussion

### Pathways Enriched in Each Pathological Stage of HCC

**FIGURE 1:**
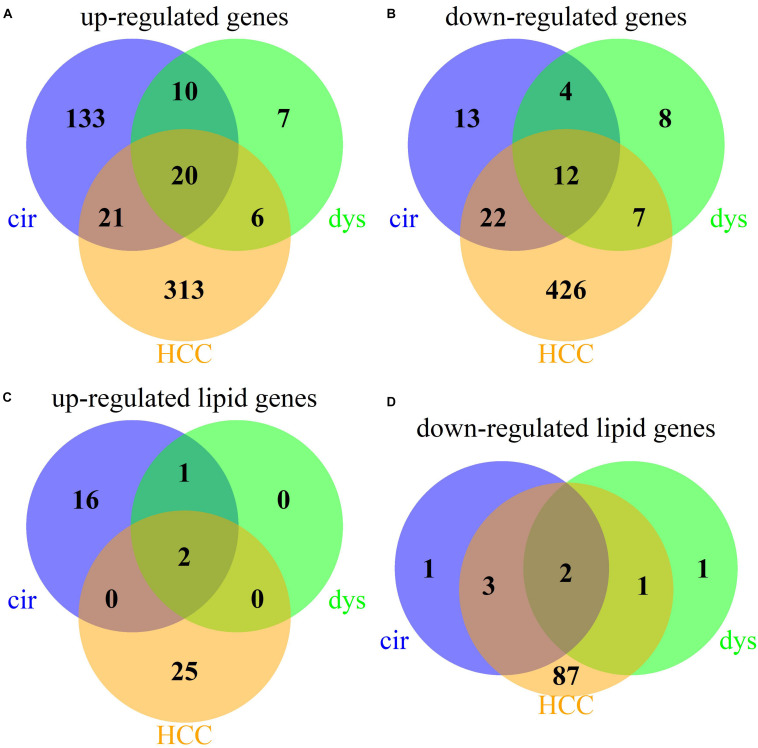
Venn diagrams for the DEGs in cirrhotic, dysplastic, and HCC samples. **(A)** Up-regulated genes. **(B)** Down-regulated genes. **(C)** Up-regulated lipid metabolic genes. **(D)** Down-regulated lipid metabolic genes.

We identified 235, 74, and 827 DEGs in cirrhotic tissues, dysplastic nodules, and HCCs, respectively, compared with the controls (Table 1). The percentage of LMGs in the DEGs at cirrhotic and dysplastic stages were both at ∼10%, but increased to 22% (180/827) in HCC (Table 1). We noted that more than half of the differentially expressed genes in dysplasia overlapped with those in the cirrhotic samples (Figures 1A,B). Two LMGs (AKR1B10 and GOLM1) were consistently up-regulated (Figure 1C) and two (CYP2C19 and DGAT2) were consistently down-regulated (Figure 1D). Among the differentially expressed LMGs, three were cirrhosis-specific up-regulated genes, eight were HCC-specific up-regulated genes, and 46 were HCC-specific down-regulated genes (Table 1 and Supplementary Table S1).

**TABLE 1:** A summary of the differentially expressed genes.

	**Cirrhotic**	**Dysplastic**	**HCC**
Up-regulated	184 (19:3)	43 (3:0)	360 (87:8)
Down-regulated	51(6:0)	31 (4:0)	467 (93:46)
Total	235(25:3)	74 (7:0)	827 (180:54)

*Values inside the parentheses are the numbers of differentially expressed LMGs and stage-specific LMGs, respectively.*

**FIGURE 2:**
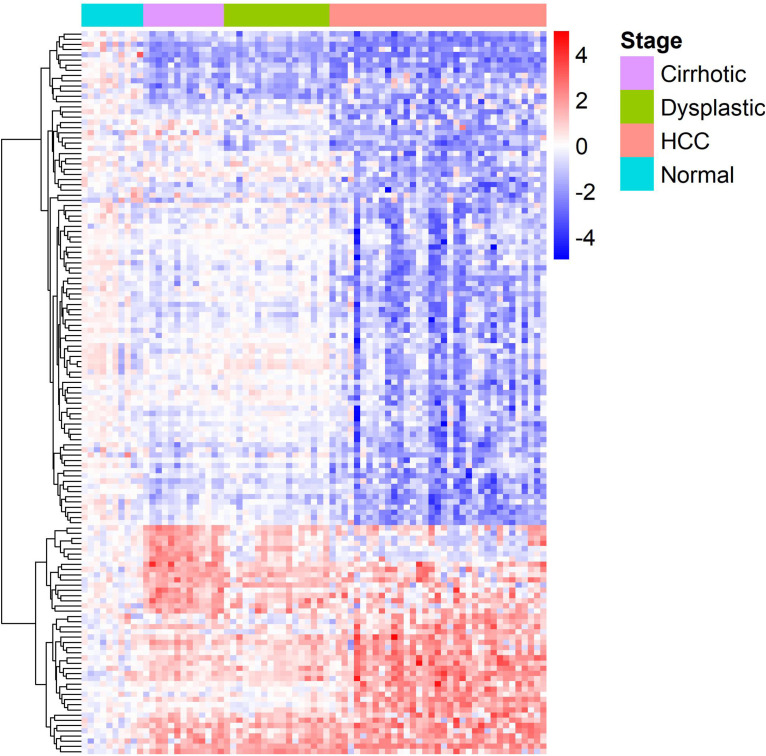
A heat-map for the differentially expressed lipid metabolic genes. Rows and columns represent genes and samples, respectively, and gene names are given in Supplementary Table S1. Expression values of each gene were centralized at the mean of normal samples and divided by the standard deviation of all samples.

The gene expression heat-map (Figure 2) shows that differentially expressed LMGs roughly fell into four groups: consistently down-regulated, HCC-specific down-regulated, HCC-specific up-regulated, and cirrhosis-specific up-regulated group which was composed of a few genes. The largest one was the HCC-specific down-regulated group.

**FIGURE 3:**
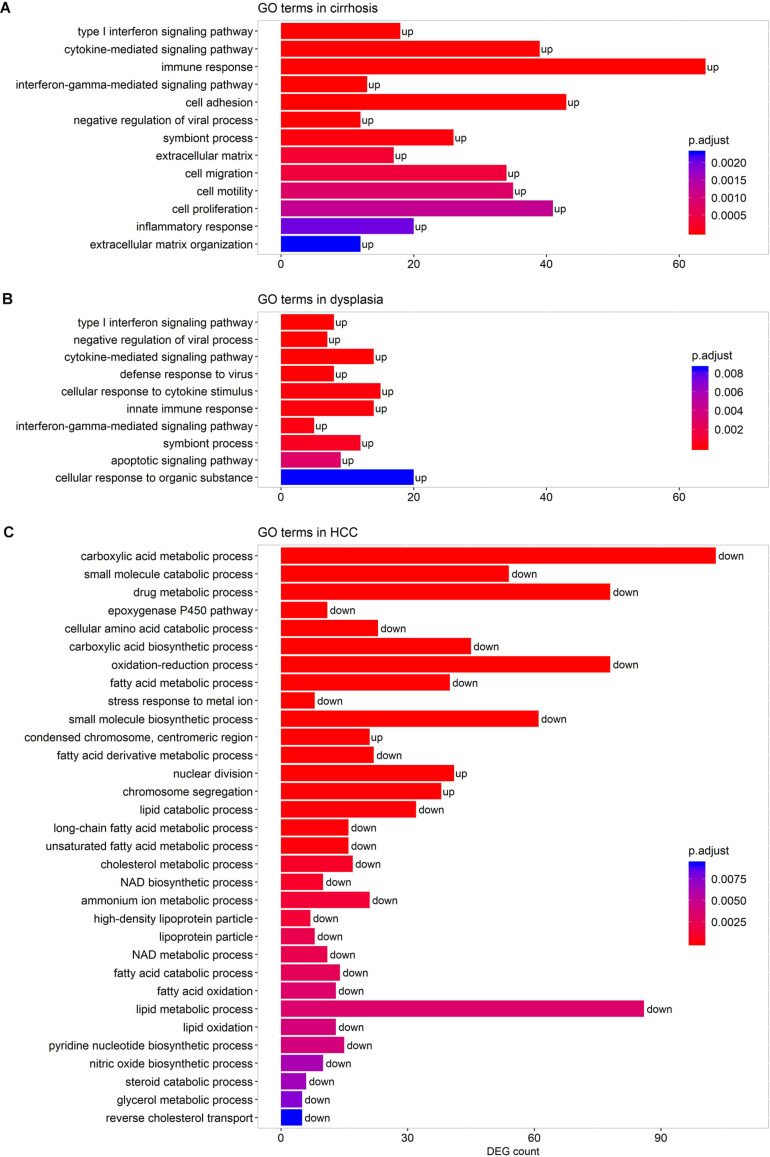
Representative GO terms enriched by the DEGs in cirrhotic **(A)**, dysplastic **(B)**, and HCC samples **(C)**. A term marked as “up” indicates that it is activated, and “down” represents that it is repressed. The list of enriched GO terms is given in Supplementary Table S2.

We performed pathway-enrichment analyses over each set of DEGs. The results, as shown in Figure 3, were: (1) in cirrhotic tissues, multiple immune responses, such as interferon gamma-mediated signaling, cytokine-mediated signaling, T cell activation, and response to virus were activated. Extracellular structure organization, cell adhesion, and cell motility were also up-regulated; (2) in dysplastic nodules, immune activity remained up-regulated as in cirrhosis, and apoptotic signaling was activated; (3) in HCC (Figure 3C), the cell cycle accelerated as indicated by the increased nuclear division and chromosome segregation. Chromosome condensation, particularly at the centromeric region, increased. While no GO terms and pathways were enriched by down-regulated genes in both cirrhotic tissues and dysplastic nodules, numerous activities, particularly the small molecule metabolic process (e.g., carboxylic acid metabolism) were substantially repressed in HCC. The lipid metabolic processes were reprogramed in HCC: both the catabolism and anabolism of lipids and fatty acids were down-regulated, compared with controls. The reduced oxidation-reduction process might have been caused by the lack of O_2_ inside the cells; and the decreased biosynthesis of NAD^+^ indicated the reduced activities of the TCA cycle and fatty acid β-oxidation (Stein and Imai, 2012; Zhu et al., 2019).

### Co-expression Network for Lipid Metabolic Genes

Pathway enrichment analyses over the DEGs in HCC revealed evident alterations in lipid metabolism in HCC. To gain knowledge about other genes that might be strongly associated with the LMGs at each stage of the disease progression, we performed co-expression analyses between the LMGs and other genes. We focused on those non-LMG genes with direct interactions with lipid metabolism, and hence analyzed only the first neighbors of LMGs in the network. We first built a co-expression network for each disease stage.

**FIGURE 4:**
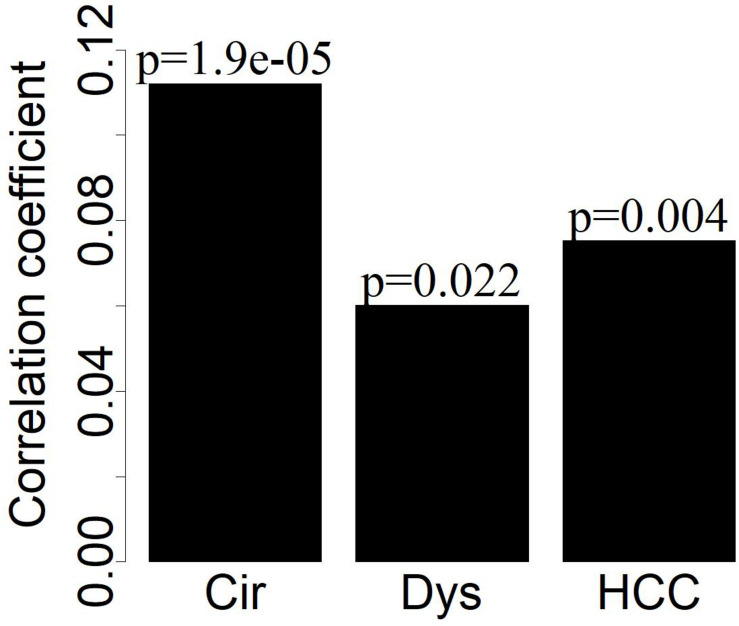
The deviation of LMGs from controls in their expression (| logFC|) has weak positive correlations (Pearson correlation) with the connectivity of LMGs.

By analyzing the co-expression networks, we noted that the extent of the differential expression of the LMGs had weak positive correlations with the connectivity of LMGs for disease samples (Figure 4), indicating that the highly differentially expressed LMGs tended to have more interactions with others.

**FIGURE 5:**
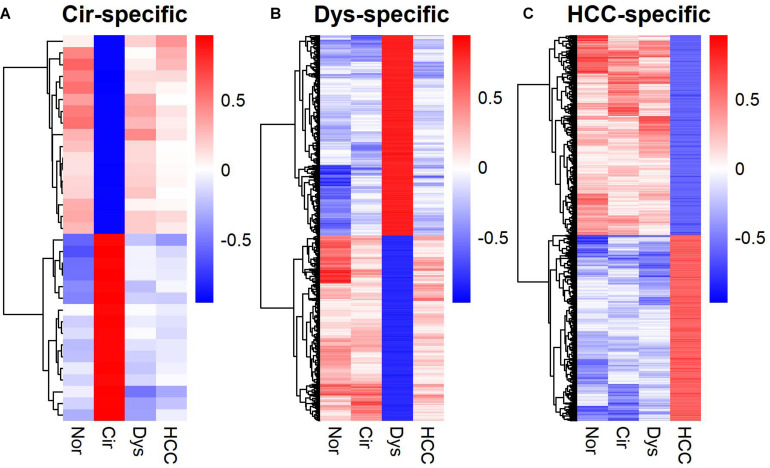
Heat-maps for stage-specific co-expression networks of lipid metabolic genes. **(A)** Cirrhotic-specific co-expression. **(B)** Dysplastic-specific co-expression. **(C)** HCC-specific co-expression. The Pearson’s correlation coefficients are color-coded as depicted in the sidebar. The co-expressed gene-pairs in the heat-maps are provided in Supplementary Table S3.

In addition to marker gene expression (Yan et al., 2020) and within-sample relative expression orderings (Zhang Z.M. et al., 2020), the gene co-expression pattern also served as a diagnostic signature. Particularly, stage-specific co-expression networks revealed important information for understanding the disease progression as well as serving as diagnostic or prognostic markers of the disease (Li et al., 2015; Xu et al., 2016; Chen et al., 2017; Liu et al., 2018; Yin et al., 2018; Zhang M. et al., 2020). Moreover, such networks, like dynamical network biomarkers, can also be used to detect the pre-disease state (Chen et al., 2012; Liu et al., 2013). We identified stage-specific co-expressions for LMGs (Supplementary Figure S1). A stage-specific co-expression refers to that occurring only in a specific stage. Hence the gene-gene correlations for a specific disease stage differ from those in the other stages (Figure 5). For the cirrhotic samples, the stage-specific co-expressions were rare and did not form a network; but for dysplastic and HCC samples, a large fraction of the co-expressed genes were connected in a group and formed a network module (Supplementary Figure S1).

**FIGURE 6:**
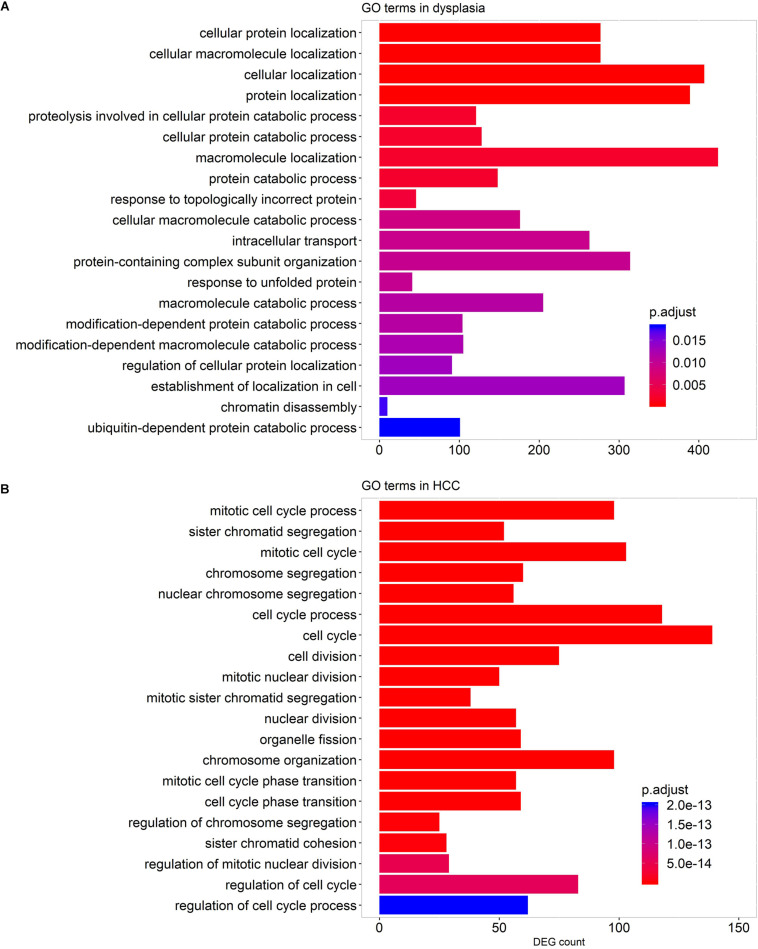
GO terms enriched by the genes co-expressed with LMGs in the stage-specific networks. Enriched GO terms for dysplastic-specific network **(A)** and HCC-specific network **(B)**. No GO terms were enriched for cirrhotic samples. The top 20 biological process-associated GO terms with small adjusted *p*-values are shown.

We performed GO term enrichment analyses to detect GO terms enriched by genes strongly co-expressed with LMGs (| *R*| > 0.7, adjusted *p*-value <0.05) in stage-specific networks. The results indicated that numerous genes co-expressed with LMGs play roles in protein localization and the catabolic process in dysplastic samples, while, in HCC samples, enriched cell cycle genes were co-expressed with LMGs (Figure 6). This implied again that cancer was distinct from pre-cancerous lesions (dysplasia) in regulating the lipid-associated process. No enriched terms were found for the cirrhotic samples.

In the stage-specific networks, nodes having high connectivity (e.g., hub genes) might be particularly important. The LMGs with the highest 10% connectivity present in all three disease stages (consistently highly connected) and the highly connected LMGs in the stage-specific networks are listed in Table 2.

**TABLE 2:** The LMGs differentially expressed in disease samples or having high connectivity in disease-specific networks.

	**DEGs**	**Highly connected LMGs^b^**
Cirrhotic-specific	Up: CFTR, ITGB8, SH3YL1	——
	Down: none	
Dysplastic-specific	Up: none	——
	Down: none	
HCC-specific^a^	Up: GPC3, PRKAA2	PLSCR4, MFSD2A, ETFDH, ESR1, HAO2, BCO2, **CYP4V2**, PTK2, **ACAT1**, HSD17B6
	Down: CYP1A2, CYP39A1, ACSM3, **LCAT**, MFSD2A, PTGS2, APOF, EGR1, **ESR1**, CETP, **RDH16**, HAO2, **MOGAT2**, APOA5, BCO2, THRSP, GBA3, SLCO1B3, CYP2B6, **ADH4**, CYP2A6, **CYP2C8**	
Consistently regulated or	Up: **AKR1B10**, **GOLM1**	ST8SIA4, **INPP4A**, CRYL1
highly connected	Down: CYP2C19, **DGAT2**	

*^*a*^Only the differentially expressed HCC-specific genes with | log_2_FC| > 2 and adjusted *p*-value <0.01 are shown, and the full gene list is given in Supplementary Table S1. ^*b*^The genes are differentially expressed LMGs (| log_2_FC| > 1 and adjusted *p*-value <0.01) having the highest 10% connectivity in stage-specific networks.*

**FIGURE 7:**
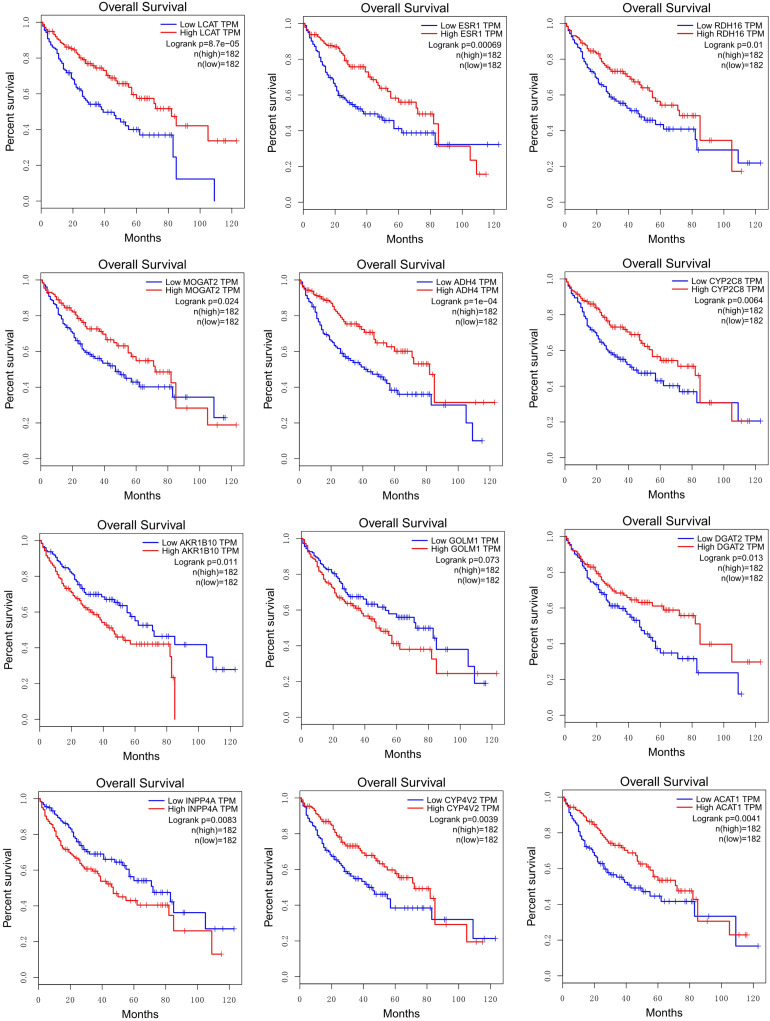
Correlations between survival rates and the expression levels of HCC-specific or consistently regulated LMGs (shown in bold in Table 2).

By performing survival analysis using GEPIA (Tang et al., 2017), we found that the expressions of some HCC-specific or consistently regulated LMGs (shown in bold in Table 2) showed significant correlations with the survival of patients (Figure 7).

Note that the microarray data of HCC analyzed here were derived from HCC samples with HCV infection, and hence the co-expression network constructed by the LMGs may represent changes of molecular interactions specific to HCV-derived HCC rather than common changes in HCC of various etiologies. It remains to be tested if the specific interactions are present in HCC of other etiologies. Although the mechanisms by which the co-expressed genes contributed to hepatocellular carcinoma are still unclear, our results deepen the understanding of HCV-originated HCC and provide useful information about the stage-specific signature for HCC.

### Reprogramed Lipid Metabolism in HCC

**FIGURE 8:**
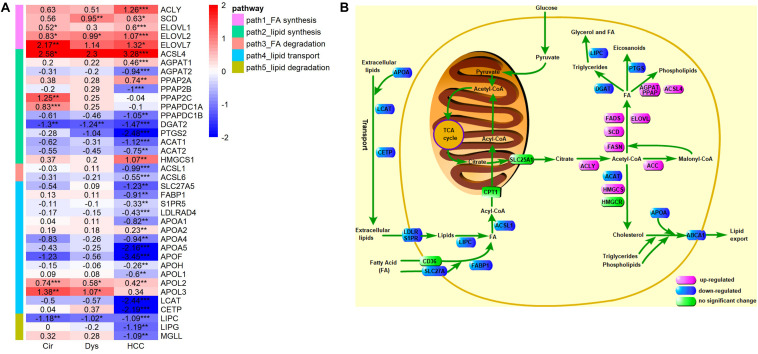
Expression levels of key LMGs in HCV-derived HCC. **(A)** A heat-map for these genes in HCC vs. controls (GSE6764), where the displayed numbers are logFC values. Only the DEGs with adjusted *p*-value <0.01 are shown, and the full list of genes analyzed here is given in Supplementary Table S1. ACSL4 is categorized into a lipid synthesis group according to its role found in (Doll et al., 2017); **(B)** A schematic depiction of the reprogramed lipid metabolism in HCC. Different colors are used to mark differential expressions: pink, blue, and green represent up-regulation, down-regulation, and no significant changes, respectively. The results of differential expression analysis and GO enrichment analysis suggest increased *de novo* synthesis of fatty acids, reduced lipid exchange between HCC tumor and its environment, and down-regulated lipid catabolic process and fatty acid oxidation in HCC.

In the above analyses, we used the following criteria to define a DEG: | logFC| > 1 and adjusted *p*-value <0.01, which may miss some differentially expressed genes. To avoid this issue, we examined the expressions of crucial LMGs particularly those encoding the enzymes that catalyze the lipid metabolism using a less stringent criterion: adjusted *p*-value <0.01. The analyzed crucial LMGs here were collected from literature (Baenke et al., 2013; Beloribi-Djefaflia et al., 2016; Cheng et al., 2018). The results, as given in Figure 8, have revealed that: (1) the *de novo* synthesis of fatty acids from citrate acids was enhanced based on the expressions of ACLY, SCD, and ELOVL in HCC (FASN was marginally up-regulated in HCC: logFC = 0.58, *p* = 0.03, adjusted *p*-value = 0.08), but not evidently up-regulated in cirrhosis and dysplasia as the key enzymes for the first and the last steps of fatty acid synthesis, ACLY and FASN, did not show differential expressions in these two disease stages; (2) down-regulation of multiple apolipoprotein genes in HCC, which can transport lipids between the liver, peripheral cells, and blood, as well as the repressed lipoprotein receptors suggest that HCC may be independent on lipids in blood circulation; the down-regulated lipid importers, FABP1 and S1PR5, also support this independence prediction; the substantial down-regulation of LCAT and CETP suggests that there is almost no reverse cholesterol transport into HCC; the slightly up-regulation of APOL2 and APOL3, apolipoproteins that may assist the movement of lipids in the cytoplasm or allow the binding of lipids to organelles, possibly suggests the requirement of lipids at subcellular locations in HCC; (3) no lipid degradation in HCC was detected, as indicated by the down-regulated lipases, LIPC, LIPG, and MGLL; (4) ACSL1 and ACSL6, enzymes that catalyze the degradation of long chain fatty acids, were down-regulated in HCC, but interestingly, another enzyme, ACSL4, of the same family was considerably up-regulated; (5) the enzyme for promoting triglyceride synthesis and downstream lipid droplet formation, DGAT2, was significantly down-regulated in cirrhosis, dysplasia, and HCC, which might be a natural consequence of the protein-energy malnutrition from the cirrhotic stage; (6) the reduced expression of PTGS2 (Cyclooxygenase 2) suggested the reduced production of prostaglandin, a pro-inflammatory signal, in HCC; (7) three enzymes, AGPAT2, PPAP2B, and PPAPDC1B, involved in phospholipid synthesis from fatty acids were down-regulated, while two other enzymes in this pathway, AGPAT1 and PPAP2A, were up-regulated; (8) the first step of cholesterol synthesis, ACAT2, was down-regulated in HCC, but the second step, HMGCS1, was up-regulated.

We also analyzed an additional microarray data (GSE14323) consisting of 19 normal, 58 HCV-cirrhotic, and 47 HCV-HCC samples to see if the results mentioned above could be rediscovered. Overall, the results derived from GSE14323 agreed well with that of GSE6764 (Supplementary Figure S2), and revealed two additional findings: (1) FABP4 and FABP5 were up-regulated in HCC; (2) EVOL6 was down-regulated in HCC.

Note that although the members of the ACSL family convert free long-chain fatty acids into fatty acyl-CoA esters, thereby playing a key role in fatty acid degradation, ACSL4 was reported to play a special role in phospholipid synthesis and ferroptosis (Doll et al., 2017). We therefore speculate that the increased expression of ACSL4 in HCC promotes the synthesis of phospholipids. Fatty acid binding proteins FABPs are thought to play roles in fatty acid uptake, transport, and metabolism (McKillop et al., 2019). FABP1 is primarily expressed in liver, while FABP4 is in adipocytes. Not only adipocyte-derived FABP4 affects cancer development, FABP4 expressed in hepatocytes was also reported to promote HCC progression (Thompson et al., 2018; McKillop et al., 2019). Consistently, we observed high expression of FABP4 in HCC. The exact mechanisms by which FABP4, FABP5, and EVOL6 function in HCC need further studies to uncover.

Our data strongly suggest a reduced lipid exchange between an HCC tumor and its environment. Hence we postulate that the lipids required for building the membrane of the proliferating cancer cell is intracellularly synthesized. Although the analysis on key enzymes is unable to give a clear picture about fatty acid degradation in HCC, our GO enrichment results clearly support that fatty acid oxidation is down-regulated in HCC.

It is worth noting that there might be strong inter- and intra-tumoral heterogeneity, which could be ascribed to (epi)genetic lesions, microenvironmental constraints, stromal interactions, and treatment effects (Park et al., 2018). Even for a specific cancer type, lipid metabolism may differ between cancer subtypes (Monaco, 2017; Koundouros and Poulogiannis, 2020). For example, receptor-positive and triple-negative breast cancers have notable differences in terms of lipid procurement, storage, and oxidation (Monaco, 2017). Therefore, we need to put special emphasis on the heterogeneity of lipid metabolism across and within tumors in cancer studies and lipid-targeted cancer therapy.

Putting these all together, we can conclude that: (1) HCC cells tend to *de novo* synthesize fatty acids and its phospholipid derivatives; (2) the lipid catabolic process and fatty acid oxidation are greatly repressed in HCC; (3) the lipid metabolism in HCC is largely independent of lipids in blood circulation; (4) the co-expression network for LMGs shows a dynamic change during the process of HCC development, and stage-specific co-expression networks for LMGs are identified; (5) several HCC-specific differentially expressed LMGs and some LMGs in HCC-specific network are associated with the cancer survival.

## Data Availability Statement

Publicly available datasets were analyzed in this study, which can be freely downloaded from GEO (https://www.ncbi.nlm.nih.gov/geo/) with the accession numbers: GSE6764 and GSE14323.

## Ethics Statement

Ethical review and approval was not required for the study on human participants in accordance with the local legislation and institutional requirements. Written informed consent for participation was not required for this study in accordance with the national legislation and the institutional requirements.

## Author Contributions

GL and YX conceived the study and wrote the manuscript. GL conducted the calculation and analysis. GjL and XC participated in the data analysis. All authors contributed to the article and approved the submitted version.

## Conflict of Interest

The authors declare that the research was conducted in the absence of any commercial or financial relationships that could be construed as a potential conflict of interest.

## Footnotes

1 http://www.geneontology.org/

## Supplementary Material

Supplementary Figure 1 Stage-specific co-expressions for LMGs. The nodes in pink and cyan represent, respectively, LMGs and genes co-expressed with LMGs.

Supplementary Figure 2 Expression levels of key LMGs in HCV-derived HCC (GSE14323) as in Figure 8A.

Supplementary Table 1 Detailed information about LMGs analyzed in this study.

Supplementary Table 2 List of GO terms enriched by the DEGs in disease samples.

Supplementary Table 3 List of stage-specific co-expressed gene-pairs.

## Abbreviations

DEG: differentially expressed gene
HCC: hepatocellular cancer
LMG: lipid metabolic gene.
