# Supplementary figures and images for: Transcriptomic Data Analyses Reveal a Reprogramed Lipid Metabolism in HCV-Derived Hepatocellular Cancer
Source: Front Cell Dev Biol. 2020 Oct 27;8:581863. doi: 10.3389/fcell.2020.581863 (PMC7652758; doi:10.3389/fcell.2020.581863)

## A Cirrhotic-specific

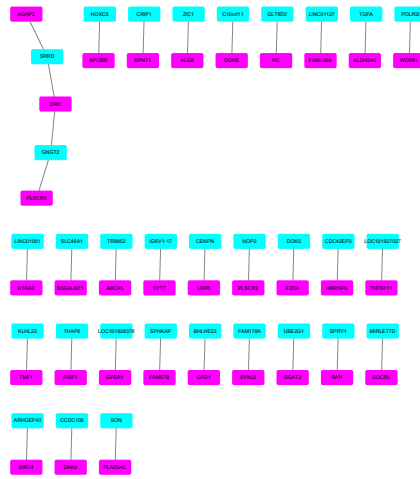

## B Dysplastic-specific

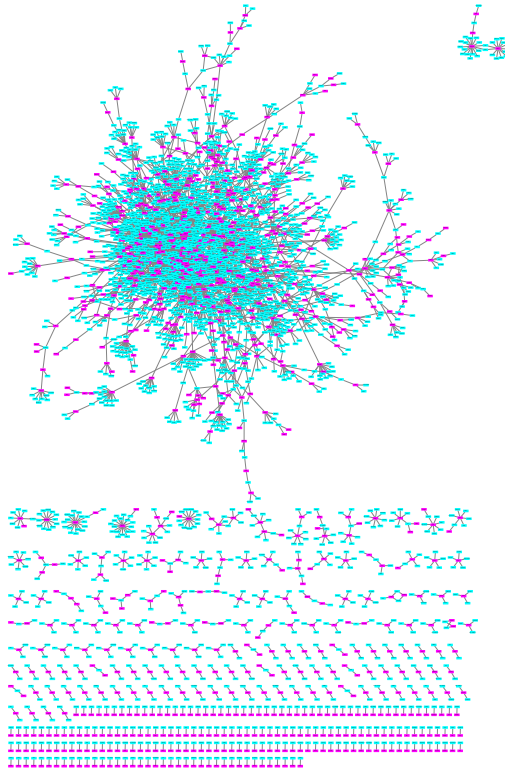

## C HCC-specific

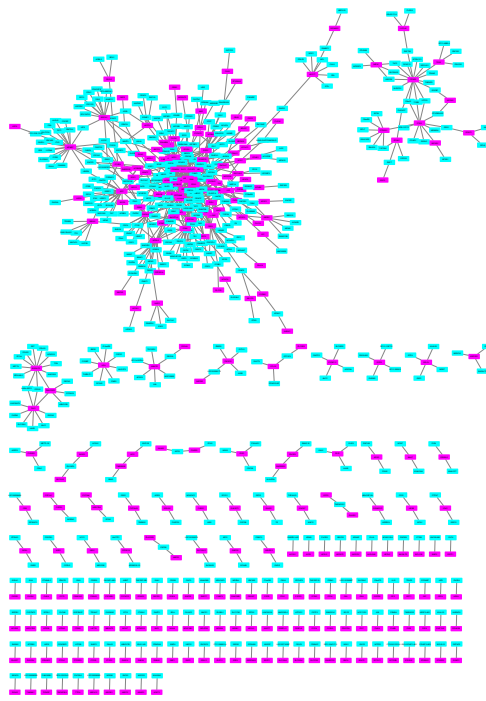

Supplement: Supplementary Figure 1 — Stage-specific co-expressions for LMGs. The nodes in pink and cyan represent, respectively, LMGs and genes co-expressed with LMGs. [file Image_1.PDF]

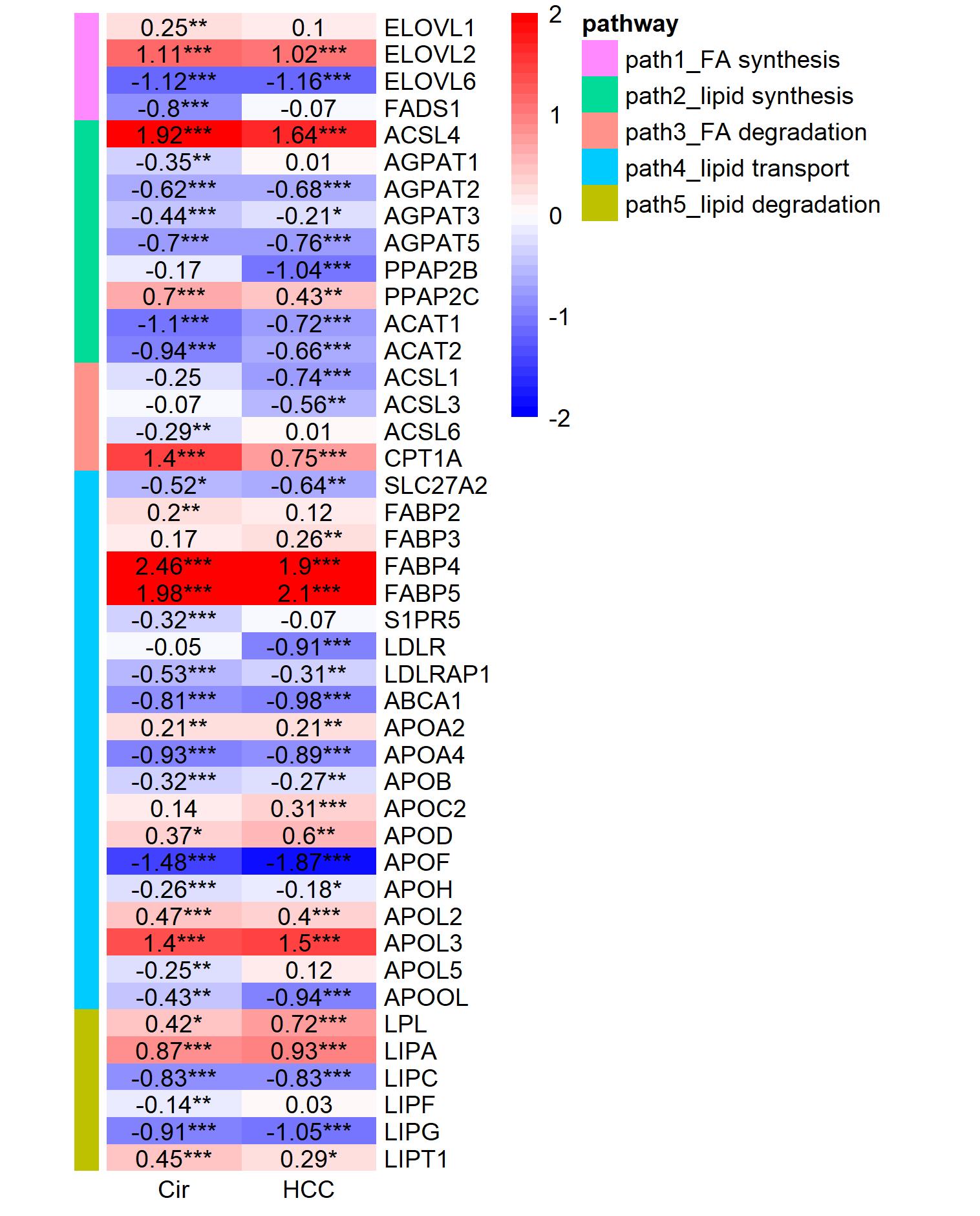

Supplement: Supplementary Figure 2 — Expression levels of key LMGs in HCV-derived HCC (GSE14323) as in Figure 8A. [file Image_2.JPEG]
